# Eosinophilic variant of chromophobe renal cell carcinoma metastasizing to the liver: Diagnostic pitfall

**DOI:** 10.1016/j.radcr.2023.07.006

**Published:** 2023-07-27

**Authors:** Daisuke Inoue, Shoji Oura

**Affiliations:** Department of Surgery, Kishiwada Tokushukai Hospital, Kishiwada-city, Osaka, 596-8522 Japan

**Keywords:** Arginase-1 immunostaining, Chromophobe renal cell carcinoma, Diagnostic difficulties, Eosinophilic variant, Metastatic liver tumor

## Abstract

An 82-year-old man developed a hypervascular renal tumor, 2 cm in size, and multiple liver tumors. Liver tumors had obscured tumor margins on ultrasonography. Positron emission tomography/computed tomography (PET/CT) showed no areas of avid radiotracer uptake in the liver. Routine pathological examination failed to demonstrate tumor cells in 9 tissue samples obtained from repeated core needle biopsies. Even a frozen section of the liver segment 8 tumor further failed to prove malignant cells, and an additive frozen section of the liver section 2 tumor finally proved atypical cells growing in tubular and solid fashions with eosinophilic cytoplasm. Tumors showed expansive growth patterns, were in direct contact with normal liver cells, had abundant micro-vessels, had only sparse hyalinized septa, and had no pale cells. Immunostaining revealed the tumor cells to be positive for CD10, CD117, and E-cadherin and negative for CK7, and PAX8, leading to the diagnosis of metastatic chromophobe renal cell carcinoma (chRCC) in the liver. Arginase-1 immunostaining clearly demarcated the boundary between the chRCC cells and normal hepatic cells. Diagnostic physicians should note that chRCCs are of low-grade malignancy despite their abundant intra-tumoral blood flow and can often pose imaging and pathologic diagnostic difficulties.

## Introduction

Solid malignancies can develop hematogenous metastasis to various distant organs such as the brain, bones, lungs, and liver. Metastatic lesions generally show similar image phenotypes to those of primary malignant lesions. Hypervascular tumors, therefore, usually develop hypervascular distant metastases regardless of their metastatic organs. It, therefore, is not so difficult to diagnose recurrence or metastasis when the primary carcinoma is evident and the presumed metastatic lesions have imaging findings similar to those of the primary tumor.

When detecting presumed distant metastatic lesions with some kind of metastasis-induced symptoms [1,2] accompanied by disease-specific tumor marker elevation, physicians often begin to treat the patient with disease-oriented therapies even when no definitive pathological diagnosis of the lesions is obtained. A tentative diagnosis of metastatic disease is readily justified by both the symptom relief and the decrease of the elevated tumor marker levels with the therapy.

The location of metastatic organs greatly affects the difficulty of tissue harvesting for pathological evaluation. Pathologic evaluation for brain lesions naturally requires a craniotomy or at least a burr hole trephination. The invasiveness of brain biopsy often leads to empiric treatment of the presumed brain metastatic lesions without their definitive pathological diagnosis. Concerning liver metastasis, ultrasound can enable physicians to easily detect liver lesions except for those adjacent to the lungs and to get tissue of the target lesions with a core needle biopsy. Physicians, therefore, often develop a therapeutic strategy for patients with presumed metastatic liver tumors, especially origin unknown, after making a pathological diagnosis by ultrasound-guided needle biopsy.

We herein report a case of metastatic chromophobe renal cell carcinoma (chRCC) to the liver having required open biopsies after pathological diagnostic failure even with the 9 core needle biopsy specimens.

## Case report

**Fig. 1:**
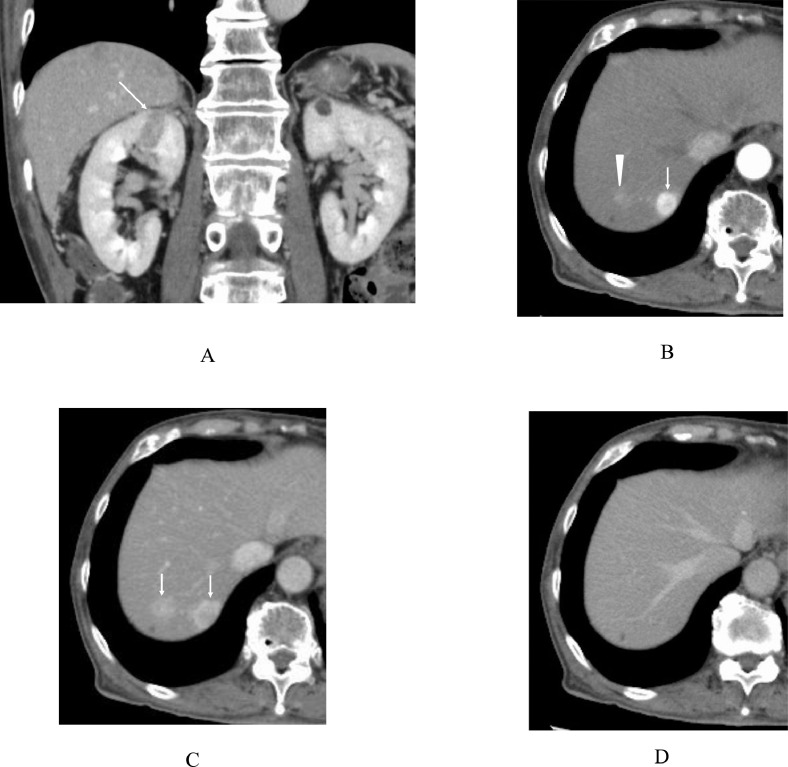
Enhanced computed tomography (CT) findings. (A) Coronal view of the enhanced CT showed an oval mass with slight enhancement in the right kidney (arrow). (B) Axial view of the enhanced CT, an arterial phase, showed round (arrow) and obscured (arrowhead) tumors with rapid enhancement in the liver. (C) Axial view of the enhanced CT, a portal phase, showed a marked washout in the liver tumors (arrows). (D) Axial view of the enhanced C, a late phase, showed complete washout in the liver tumors.

**Fig. 2:**
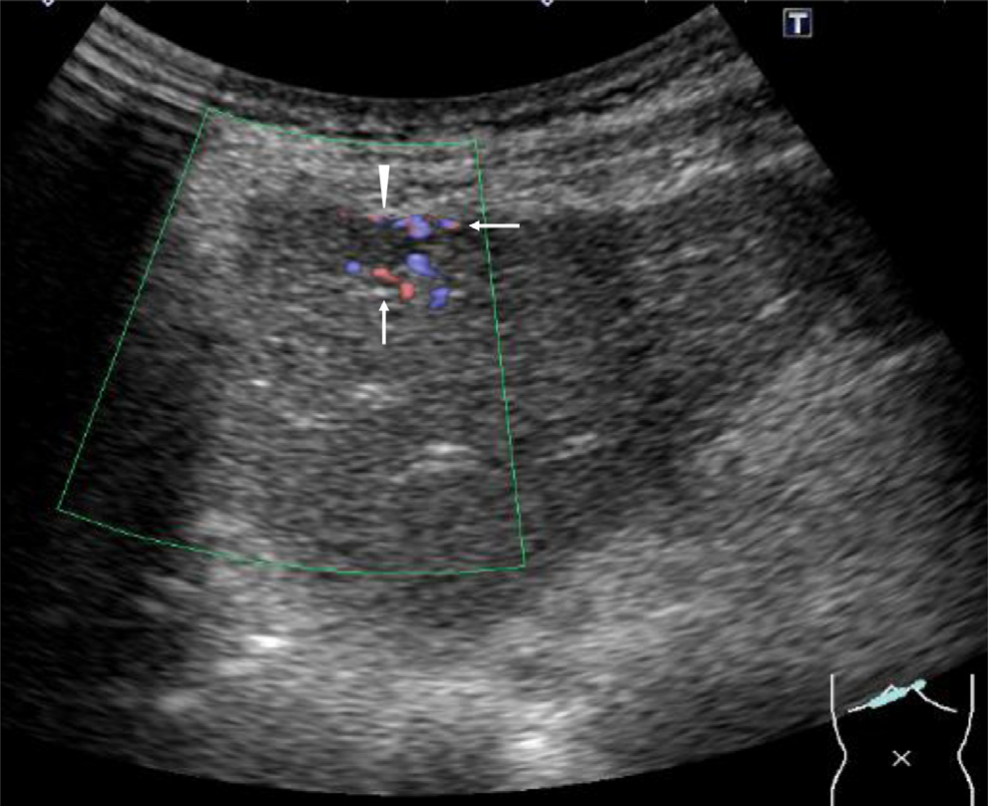
Ultrasonography showed a presumed tumor (arrowhead) with obscured margins and abundant vascularity (arrows).

**Fig. 3:**
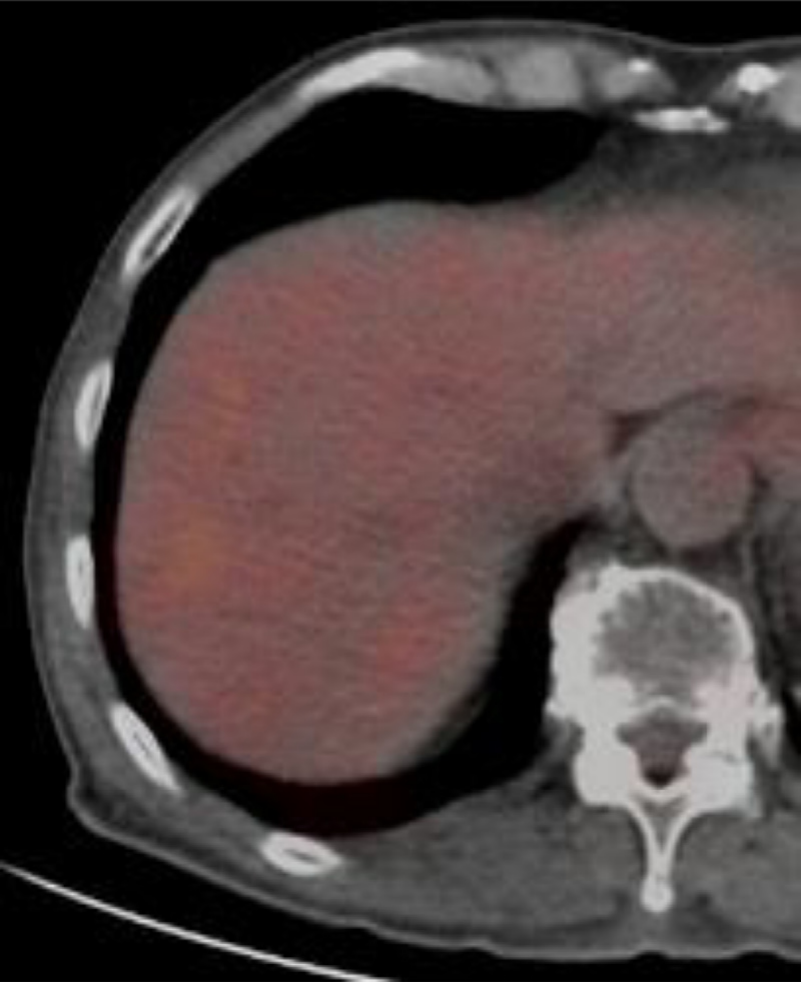
Positron emission tomography (PET) /CT PET/CT showed no fluorodeoxyglucose accumulation in the liver.

**Fig. 4:**
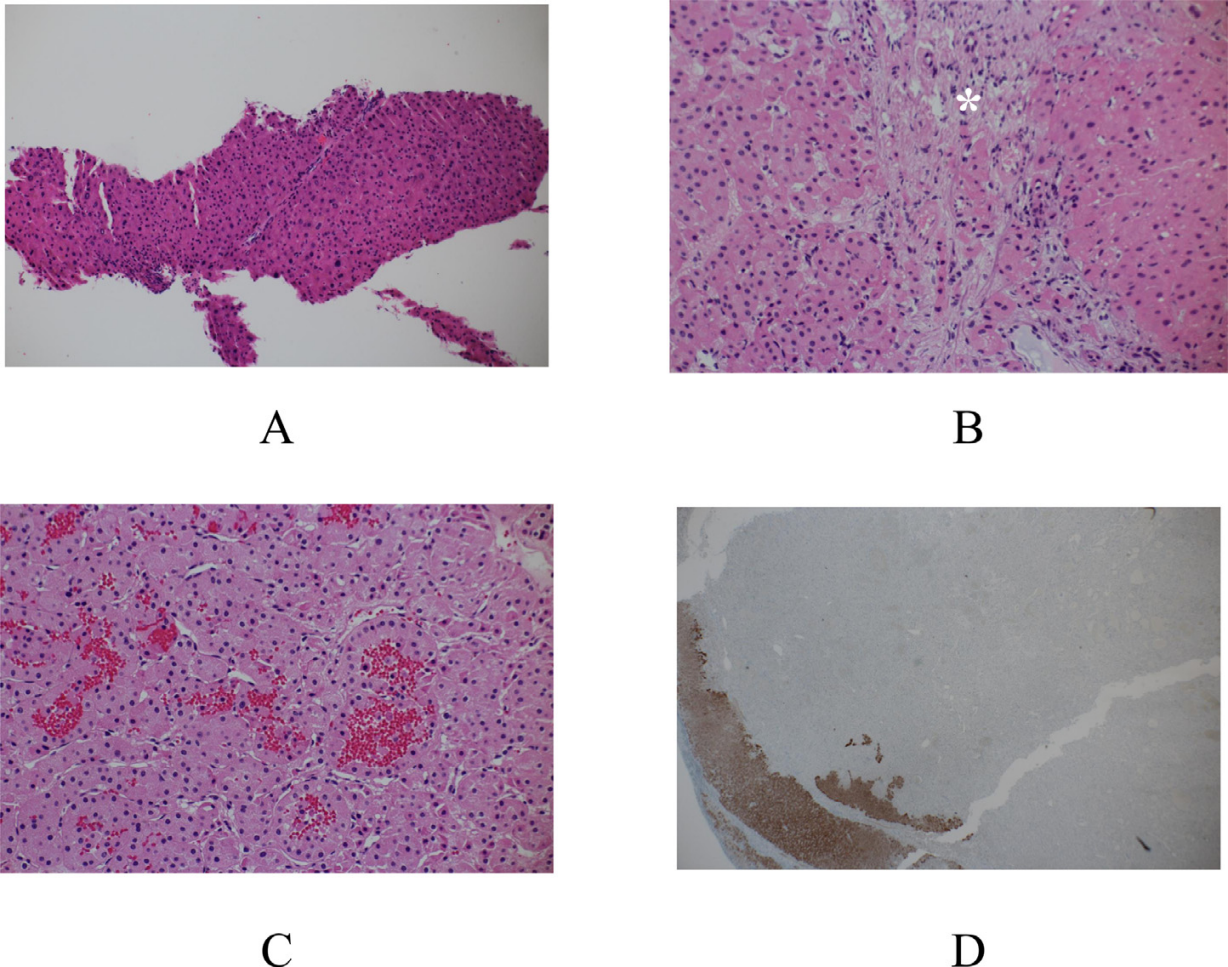
Pathological findings. (A) Pathological study of the core needle biopsy specimens showed no atypical cells either with nuclear polymorphism or with mitoses. (B) A magnified view of the resected specimen showed that hyalinized vascular septa (asterisk) separated the normal hepatic cells (right side) and atypical cells (left side). (C) A highly magnified view of the tumor area contained vascular-rich area. (D) Arginase-1 immunostaining clearly demarcated the normal hepatic cells (brownish area) and tumor cells.

An 82-year-old man complaining of hematochezia was referred to our hospital. After the pathological diagnosis of rectal cancer with colonoscopic biopsy of the rectal tumor, positron emission tomography/computed tomography (PET/CT) for preoperative staging showed fluorodeoxyglucose (FDG) accumulation both in the rectum and prostate. The patient, therefore, simultaneously underwent a biopsy of the prostate tumor and robot-assisted anterior rectal resection for the rectal cancer under general anesthesia. Pathological study showed that the prostate tumor was an adenocarcinoma with a Gleason score of 7 and the rectal cancer was a well-differentiated adenocarcinoma without regional lymph node metastasis. The patient, therefore, was followed-up without adjuvant chemotherapy for rectal cancer and with combined androgen blockade therapy using bicalutamide and leuprorelin for prostate cancer. Seven months after the operation, follow-up contrast-enhanced CT showed an encapsulated kidney tumor with a washout pattern, 2 cm in size, and multiple liver tumors with a similar time-intensity signal pattern. The latter tumors had a ring enhancement in the early phase and a complete washout in the late phase, highly suggesting those to be metastatic liver tumors (Fig. 1). Ultrasonography showed multiple oval and iso-echoic masses with obscured tumor margins (Fig. 2). PET/CT at this time point also showed no FDG accumulation in the liver (Fig. 3). Under the tentative diagnosis of renal cell carcinoma (RCC) with multiple liver metastases, we did core needle biopsy to the liver lesions. Three core needle biopsy specimens showed no atypical cells pathologically (Fig. 4). A repeated contrast-enhanced CT after the first core needle biopsy showed slight enlargement of the liver tumors. Another attempt at tissue sampling through core needle biopsy was made. Six core needle biopsy specimens, however, again showed no malignant findings. Highly suspected pathological under-diagnosis, judged by image findings, forced us to get tumor tissue by open biopsy. Even a frozen section further showed no malignant findings in the tumor resected from the liver segment 2. We, therefore, resected another liver tumor in segment 8. Additive frozen section finally showed atypical cells growing in tubular and solid fashions with eosinophilic cytoplasm, leading to the diagnosis of metastatic RCCs in the liver. A permanent section of the resected 2 lesions showed eosinophilic atypical cells, suggesting the false negative results of the segment 2 tumor on frozen section. Immunostaining revealed the tumor cells to be positive for CD10, CD117, and E-cadherin and negative for CK7, PAX8, and Arginase-1 (Fig. 4). These findings eventually brought us to the definitive diagnosis of eosinophilic variant of chromophobe RCC (chRCC). The patient began to receive sunitinib [3] as the first line treatment for metastatic chRCC but showed poor compliance to the drug due to its side effects, resulting in early cessation of the therapy followed by image evaluation without any treatments. Due to slight tumor growth in 2 months on enhanced CT, the patient has been receiving nivolumab monotherapy [4] for 4 months without any tumor growth or regression.

## Discussion

chRCC is a relatively rare variant of RCC and shows pathological typical findings such as prominent cell membranes, wrinkled nuclei with perinuclear haloes, and pale to eosinophilic cytoplasm. In addition, tumor cells are generally separated by incomplete, often hyalinized, vascular septa [5]. The pale cells usually surround the eosinophilic cells peripherally. In this case, although prominent cell membranes were observed, inconspicuous perinuclear haloes, uniform small cells with fine oxyphilic granularity, and the absence of pale cells easily led us to the pathological under-diagnosis of the liver lesions even by a frozen section on open biopsy. Furthermore, it was more difficult to detect prominent cell membranes due to cell overlapping caused by thick section preparation from the small core needle specimens.

In this case, tumors showed expansive growth pattern, were in direct contact with normal liver cells, had abundant micro-vessels, and had only sparse hyalinized septa. It is well known that acoustic impedance of the blood is similar to that of liver parenchyma. This fact and pathological findings observed in this case well explain the obscured tumor borders on ultrasonography due to less ultrasonographic reflection based on the similarity of acoustic impedance among tumor cells, blood-filled micro-vessels, and surrounding normal hepatic cells. This obscured tumor borders and relatively small sizes of liver tumors might have negatively affected the accuracy of core needle biopsies.

Tumor cells, especially eosinophilic cells, of chRCC extremely resembled to those of normal hepatic cells morphologically, causing the difficult detection of metastatic tumors on plain CT due to the similarities of X-ray attenuation coefficients between tumor cells and normal hepatic cells. Consecutive 2 PET/CTs without contrast medium also failed to clarify the presence of multiple metastatic liver tumors. Conversely, CT and MRI with contrast medium clearly showed hyper-vascular and hyperintense tumors both with a rapid washout pattern. These facts suggest that the metastatic liver tumors of chRCC are of low-grade malignancy despite their abundant intratumoral blood flow. Generally speaking, the more vascularity a tumor has, the more aggressive nature it has. This feature might be attributed to the characteristics of the organs that developed this disorder. Kidneys have the function of removing waste products from the body by filtering a large amount of blood. Kidneys, therefore, are basically hyper-vascular organs, and the vast majority of kidney tumors are also hypervascular tumors. Eosinophilic variant of chRCC should be understood as a tumor that is essentially hyper-vascular [6] but shows indolent biology.

Probably due to their indolent biology, the average size of chRCCs is relatively large at 7 cm at the time of diagnosis in the literature [[5], [7]]. The size of the presumed primary chRCC detected in the right kidney was small as 2cm in this case. There is no doubt that the incidental detection of chRCC on imaging evaluation for rectal cancer reflects the small size of the tumor. It, however, is unclear why such a small chRCC had caused multiple liver metastases. These facts might suggest that the eosinophilic variant of chRCC can easily spread to distant organs based on its hypervascular nature but might not directly imply the deteriorated overall survival even when developing distant metastasis.

## Conclusion

Eosinophilic variant of chRCC metastasizing to the liver often causes pathological and image diagnostic difficulties. Clinicians should be familiar with this disorder to avoid unnecessary diagnostic approaches.

## Patient consent

Written informed consent was obtained from the patient for the publication of this case report and any accompanying images.

## Author contribution

DI designed the concept of this study. SO drafted the manuscript.
